# Early neurotransmission impairment in non-invasive Alzheimer Disease detection

**DOI:** 10.1038/s41598-020-73362-z

**Published:** 2020-10-02

**Authors:** Carmen Peña-Bautista, Isabel Torres-Cuevas, Miguel Baquero, Inés Ferrer, Lorena García, Máximo Vento, Consuelo Cháfer-Pericás

**Affiliations:** 1 Neonatal Research Unit, Health Research Institute La Fe, Avda de Fernando Abril Martorell, 106; 46026 Valencia, Spain; 2 Neurology Unit, University and Polytechnic Hospital La Fe, Valencia, Spain

**Keywords:** Neurochemistry, Biochemistry, Biomarkers, Diseases, Alzheimer's disease

## Abstract

Alzheimer Disease (AD) is a pathology suffered by millions of people worldwide and it has a great social and economic impact. Previous studies reported a relationship between alterations in different amino acids and derivatives involved in neurotransmission systems and cognitive impairment. Therefore, in this study the neurotransmission impairment associated to early AD has been evaluated. For this purpose, different amino acids and derivatives were determined in saliva samples from AD patients and healthy subjects, by means of an analytical method based on chromatography coupled to tandem mass spectrometry. Results showed statistically significant differences in salivary levels for the compounds myo-inositol, creatine and acetylcholine; and other compounds (myo-inositol, glutamine, creatine, acetylcholine) showed significant correlations with some cognitive tests scores. Therefore, these compounds were included in a multivariate analysis and the corresponding diagnosis model showed promising indices (AUC 0.806, sensitivity 61%, specificity 92%). In conclusion, some amino acids and derivatives involved in neurotransmission impairment could be potential biomarkers in early and non-invasive AD detection.

## Introduction

Alzheimer Disease (AD) is a pathology suffered by millions of people worldwide and the increasing number of cases in recent years generates a great concern for the economic and social effects produced^1,2^. In addition to this, there is a concern about the still little knowledge about the mechanisms involved in the onset and development of the disease, besides there is a lack of early diagnostic methods, as well as effective treatments to fight against the disease^3–5^.

AD is a long neurodegenerative disease that produces different anatomical and physiological changes not only in brain but also in periphery^6–9^. In the course of the disease, some peptides aggregates appear in the brain, they are i) amyloid plaques, generated by the accumulation of β-amyloid peptide^10^ and ii) neurofibrillary tangles, generated by tau hyperphosphorylation or acetylation^11,12^. Physiologically, a reduction of synapsis occurs^13^, and neurotransmission is altered. In this sense, the study of different compounds involved in neurotransmission systems could be useful^14^. Previous studies have reported relationship between different neurotransmitters and AD pathology^15^, most of them used plasma or CSF samples^16–18^, while only a few used non-invasive samples, such as urine from murine animal models^19,20^.

Specifically, acetylcholine (Ach) is the most studied neurotransmitter in AD. In fact, its impairment constitutes one of the most studied therapeutic targets^21^. In addition, myo-inositol has been evaluated in AD brain by means of Magnetic Resonance Spectroscopy (MRS)^22^. Moreover, Kuzyk et al. found creatine accumulations in AD mouse model^23^. In addition, CSF glutamate and glutamine were found elevated in probable AD patients^24^. Definitely, different amino acids such as glutamate, serine and alanine seem to play an important role in AD cognition decline^25^. However, this is the first study that evaluate the salivary levels of different related-neurotransmission compounds in AD patients. In general, saliva sampling and processing involve some preanalytical variables that should be taken into account to obtain reproducible results among studies^26^.

The aim of this study is to evaluate some amino acids and derivatives related to neurotransmission alterations under AD conditions as potential biomarkers for early and non-invasive AD detection.

## Material and methods

### Participants selection and samples collection

The study participants were patients from the Neurology Service in the University and Polytechnic Hospital La Fe (Valencia, Spain) who previously signed the informed consent. The experimental protocol for the study was approved by the Ethics Committee (CEIC) of the Health Research Institute La Fe (Valencia, Spain), and it is in accordance with the appropriate guidelines (Declaration of Helsinki).

Participants were classified into AD (including mild cognitive impairment (MCI) due to AD (n = 17) and mild to moderate dementia due to AD (n = 14) and healthy control (HC, (n = 12)) groups. For this, they were subjected to neuropsychological tests (Repeatable Battery for the Assessment of Neuropsychological Status (RBANS), Clinical Dementia Rating (CDR), Minimental State Examination (MMSE), Functional Activities Questionnaire (FAQ))^27–30^, structural neuroimaging by means of nuclear magnetic resonance (NMR) or computerized axial tomography (CAT)^31^, and cerebrospinal fluid (CSF) biomarkers (β-amyloid peptide (Aβ), total Tau (t-Tau), phosphorylated Tau (p-Tau))^32,33^. Specifically, in the AD group, the MCI-AD participants showed cognitive complaints without daily living activities impairment, while mild dementia-AD participants showed minor daily living activities impairment. This classification was carried out following the National Institute on Aging-Alzheimer’s Association (NIA-AA) recommendations^34–36^. Also, all AD participants showed positive levels of CSF biomarkers (Aβ < 700 pg mL^-1^, t-Tau > 380 pg mL^-1^, p-Tau > 70 pg mL^-1^) and altered neuropsychological evaluation (CDR > 0, RBANS.DM < 65, MMSE < 27). However, the HC group was characterized by negative CSF biomarkers, and normal neuropsychological evaluation. Regarding exclusion criteria, patients with a history of structural brain disease (tumour, stroke, etc.), major head trauma, epilepsy, multiple sclerosis and major psychiatric disorders were excluded, as well as patients not able to undergo neuropsychological evaluations.

Saliva samples were obtained from all the participants. They were whole-mouth saliva and collected by spitting into sterile bottles (without additives) between 10 and 12 a.m. (minimum 30 min after breakfast). Participants rinsed their mouth before saliva collection, between 1–2 mL could be collected across subjects with minor differences between control and case subjects. Then, the samples were aliquoted into 2 mL tubes, and those with visible blood contamination were excluded from the study. Finally, samples were stored at -80ºC until the analysis.

### Standards

Glutamate, glutamine, γ-aminobutyric (GABA), taurine (Taur), aspartic acid, myo-inositol (MI), N-Acetyl-L-aspartic acid (NAA), aspartic acid (AA), creatine (Cr), acetylcholine (Ach) and acetonitrile were obtained from Sigma-Aldrich (St. Louis, MO, USA). Deuterated phenylalanine (Phe-D5) with a 98% atom D enrichment was purchased from CDN Isotopes (Pointe-Claire, Canada).

### Sample treatment

The saliva sample (150 µL) was added to 300 µl of acetonitrile and centrifuged at 1200 g, 5 min at 4ºC. Then, 5µL of the internal standard (IS) solution (Phe-D5, 10 mmol L^-1^) were added to 95 µL of supernatant. The IS is an amino acid added as corrector of injection volume and ionization procedure. The range of calibration to quantify the analytes in samples was 0.3–5000 µmol L^-1^. Finally, the samples were injected in the Ultra Performance Liquid Chromatography–tandem Mass Spectrometry (UPLC-MS/MS) system. Simultaneously, salivary total proteins were determined in each sample (5 µL) using a colorimetric protein assay kit (Pierce BCA) to standardize the analytes concentrations results. In general, these concentrations vary depending on saliva flow. So, the use of total proteins as corrective index approximates salivary flow rate adjustment^37,38^.

### UPLC-MS/MS analysis

The chromatographic system was a Waters Acquity UPLC-XevoTQ system (Milford, MA, USA) with triple quadrupole as mass analyzer. The instrumental conditions were positive electrospray ionization (ESI), capillary voltage 3.50 kV, extractor 5.00 V, source temperature 120 °C, desolvation temperature 350 °C, nitrogen cone flow 50 L/h and desolvation gas flow 750 L/h.

Separation conditions were selected to achieve appropriate chromatographic retention and resolution by using an HILIC column (100 × 2.1 mm, 7 µm, 100 Å) from Phenomenex. Mobile phase used was CH3OH (5 mmol L^-1^ NH4HCO2): H2O (5 mmol L^-1^ NH4HCO2), (70:30) with isocratic gradient 10 min. The flow rate, column temperature and injection volume were set at 0.4 mL/min, 30°, and 5 µL, respectively.

During batch analysis, samples were kept at 4 °C. Mass spectrometric detection was carried out by multiple reaction monitoring (MRM) (see Table 1) (all 10 compounds analysed in a single MRM run).

**Table 1:** MS/MS acquisition parameters.

Analyte	Parent ion (M/Z)	Cone (V)	Daughter ion (M/Z)	Confirmation ion	Collision energy (Ev)
Glutamate	148	20	84	102	15
Glutamine	147	10	84	130	10
GABA	104	20	69	87	10
Aspartic acid	134	20	74	88	10
Acetylcholine	146	20	87	–	10
Creatine	132	20	90	–	10
NAA	176	15	134	158	10
Myo-inositol	179	20	161	–	10
Taurine	126	15	108	–	10
Phe-D5	171.1	35	125	–	10

NAA: N-Acetyl-L-aspartic acid.

The data were acquired and processed using the MassLynx 4.1 and QuanLynx 4.1 (Waters) software, respectively. Peak area integration was used, and analytes responses were expressed as ratios to the Phe-D5 (IS) in all standards and samples.

### Method validation

The validation procedure consisted of the assessment of some analytical characteristics (linearity, precision, accuracy, limit of detection (LOD), limit of quantification (LOQ), stability). The individual standards were prepared in H_2_O, and the calibration standards in CH_3_CN:H_2_O (50:50, v/v). Standards were run in triplicate. The linearity was evaluated constructing a calibration curve (0.1–5000 nmol L^-1^) for each analyte (n = 9). The precision was estimated from standards at mid concentration level (1000 nmol L^-1^ for each analyte) within one validation batch (intra-day) and among validation batches (inter-day, along 1 month) (by triplicate). The accuracy was evaluated by means of the recovery test. For that, saliva samples were spiked at three concentration levels (low, mid, high), and they were analyzed each of the three validation days. The LODs and LOQs were established, as the concentrations generating a signal-to-noise ratio of 3and 10, respectively. Analytes stability after three freeze–thaw cycles was assessed by means of a spiked saliva sample (1000 nmol L-1, each analyte).

### Statistical analysis

Univariant analysis was carried out using SPSS software version 20.0 (SPSS, Inc., Chicago, IL, USA), and for the multivariant analysis Unscrambler software v7.6 (Norway) was used. Differences between AD and healthy control participants were evaluated by means of t-test for numerical variables with normal distribution (Kolmogorov–Smirnov test), expressing results as mean ± standard deviation. While, Mann–Whitney and Chi-square non-parametric tests were used for numerical and categorical variables without normal distribution, expressing results as median and interquartile range (IQR). In addition, correlations between salivary analytes and standard neuropsychological scales, as well as CSF biomarkers were analysed by Pearson Correlation. In all the cases, statistical significance was fixed in a *p* value of 0.05.

Multivariate analysis was carried out by means of Partial Least Square (PLS) using some dependent variables (metabolites) and one independent variable (participant’s group). Then, the Receiver operating characteristic curve (ROC) of the model was obtained.

## Results

### Analytical performance data

The analytical method was validated by employing standards containing all the analytes in the 0.1–5000 nmol L^-1^ concentrations range. Results are summarized in Supplementary Material (Table S1). The method provided an adequate linearity for all the analytes (R^2^ between 0.996 and 0.999), and satisfactory sensitivity (LODs between 0.09 and 1.4 nmol L^-1^). Also, suitable precision was obtained with intra-day and inter-day coefficients of variation of 0.8–3.2% (n = 3) and 2.9–10.2% (n = 6), respectively (at a concentration of 1000 nmol L^-1^). The accuracy of the method was evaluated by analysing spiked saliva samples containing analytes at different concentrations (low, medium and high) within the tested concentration ranges. The results obtained are listed at Table S2 (Supplementary Material), quantitative recoveries were achieved for all the analytes. The analytes stability was assayed after three freeze thaw cycles, determining the concentrations in spiked saliva samples (1000 nmol L^-1^, each analyte) by triplicate. The recoveries were between 88 and 106%. So, no significant deterioration of the analytes was observed.

### Demographic and clinic participants description

Participants’ demographic and clinical data are shown in Table 2. As it is expected, statistically significant differences were observed between participants groups for neuropsychological variables (RBANS, CDR, FAQ, MMSE) and CSF biomarkers (β-amyloid, Tau, p-Tau).

**Table 2:** Demographic and clinical variables of the study participants.

	HC (n = 12)	AD (n = 31)	*p* Value
Age (years (median, IQR))	69 (60, 70)	69 (67, 74)	0.072
Gender (female, n (%))	4 (33%)	18 (58%)	0.146
CSF β-Amyloid (pg mL^-1^) (median (IQR))	1178 (1031, 1414)	552 (465, 678)	< 0.001*
CSF total Tau (pg mL^-1^) (median (IQR))	274 (167, 368)	641 (387, 1043)	0.002*
CSF phosphorylated Tau (pg mL^-1^) (median (IQR))	44 (36, 57)	101 (71, 146)	< 0.001*
CDR (median, IQR)	0 (0, 0)	0.5 (0.5, 1)	< 0.001*
MMSE (median, IQR)	30 (28, 30)	24 (18, 26)	< 0.001*
RBANS.MI (median, IQR)	86 (81, 95)	53 (40, 73)	< 0.001*
RBANS.VC (median, IQR)	201 (81, 116)	72 (56, 89)	0.001*
RBANS.L (median, IQR)	92 (83, 95)	60 (51, 82)	< 0.001*
RBANS.A (median, IQR)	97 (84, 100)	60 (56, 79)	< 0.001*
RBANS.DM (median, IQR)	98 (89, 105)	48 (40, 60)	< 0.001*
FAQ (median, IQR)	0 (0, 1)	6 (1, 13)	< 0.001*

*p < 0.05.

IQR: Interquartile range, CDR, clinical dementia rating, RBANS: repeatable battery for the assessment of neuropsychological status (immediate memory [RBANS.IM], visuospatial/constructional [RBANS.V/C], language [RBANS.L], attention [RBANS.A], delayed memory [RBANS.DM]). FAQ: Functional Activities Questionnaire.

### Amino acids and derivatives impairment in Alzheimer Disease

**Figure 1:**
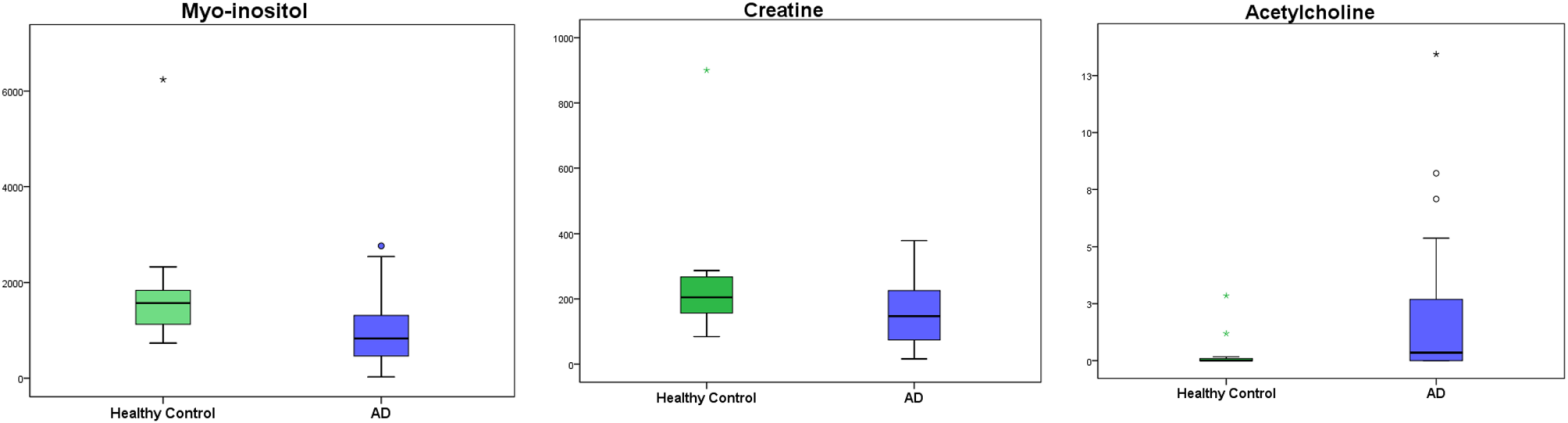
Box plot representing the myo-inositol creatine and acetylcholine salivary levels found for each participant group.

Different amino acids and derivatives (aspartic acid, glutamic acid, glutamine, GABA, creatine, taurine, N-acetil aspartate, myo-inositol, acetylcholine) were determined in saliva samples from AD patients at early stages of the pathology (MCI and mild dementia), and from HC participants (Table 3). As result, salivary myo-inositol (p = 0.018) and creatine (p = 0.049) showed lower levels in AD compared to control group, while acetylcholine showed higher levels in AD patients (p = 0.015) (Fig. 1). Not statistically significant differences were found for the other compounds. Also, the compounds levels were analysed in function of gender, observing that only glutamine showed statistically significant differences (p = 0.031).

**Table 3:** Concentrations of neurotransmitters in saliva samples.

	Healthy control (n = 12)^a^	AD (n = 31)^a^	*p* Value
Taurine (ng mg protein ^-1^)	3453.71 (1248.69)	3834.07 (2409.42)	0.607
NAA (ng mg protein ^-1^)	95.38 (60.86)	80.70 (64.73)	0.502
Myo-inositol (ng mg protein ^-1^)	1867.83 (1451.67)	1027.38 (776.03)	0.018*
Aspartic Acid (ng mg protein ^-1^)	124.18 (77.57)	169.38 (138.75)	0.186
Glutamic Acid (ng mg protein ^-1^)	953.14 (392.68)	1349.75 (1072.05)	0.083
Glutamine (ng mg protein ^-1^)	1525.73 (1518.57)	3582.30 (3544.51)	0.060
Creatine (ng mg protein ^-1^)	255.17 (213.58)	157.47 (103.85)	0.049*
GABA (ng mg protein ^-1^)	8.94 (4.91)	12.41 (18.30)	0.524
Acetilcholine (ng mg protein ^-1^)	0.02 (0.03)	1.91 (3.13)	0.015*

*p < 0.05.

^a^Mean (standard deviation).

After that, some correlations were observed between some salivary analytes and standard neuropsychological scales in AD. Specifically, delayed memory domain from RBANS scale (RBANS.DM) correlated with myo-inositol (PCC = 0.327, p = 0.032) and acetylcholine levels (PCC = -0.304, p = 0.047); as well as MMSE score correlated with myo-inositol (PCC = 0.437, p = 0.003), glutamine (PCC = -0.337, p = 0.027) and creatine (PCC = 0.342, p = 0.025) levels (see Figure S1 in Supplementary Material). Moreover, we found that salivary myo-inositol levels correlated with CSF β-amyloid (PCC = 0.351, p = 0.028).

Then, different diagnosis models were developed to compare individual accuracy of the selected compounds, as they showed statistically significant differences between AD and HC groups, as well as correlations with some relevant clinical variables used in AD diagnosis. Results indicated that acetylcholine (AUC-ROC 0.660 (CI 95%, 0.492–0.828) and glutamine (AUC-ROC 0.777 (CI 95%, 0.619–0.935) showed satisfactory accuracy; while creatine (AUC-ROC 0.331 (CI 95%, 0.167–0.494) and myo-inositol (AUC-ROC 0.261 (CI 95%, 0.113–0.408) showed poor accuracy. Finally, a multivariate model was performed including all these compounds (myo-inositol, creatine, glutamine, acetylcholine) (p ≤ 0.06).

### Multivariate analysis.

**Figure 2:**
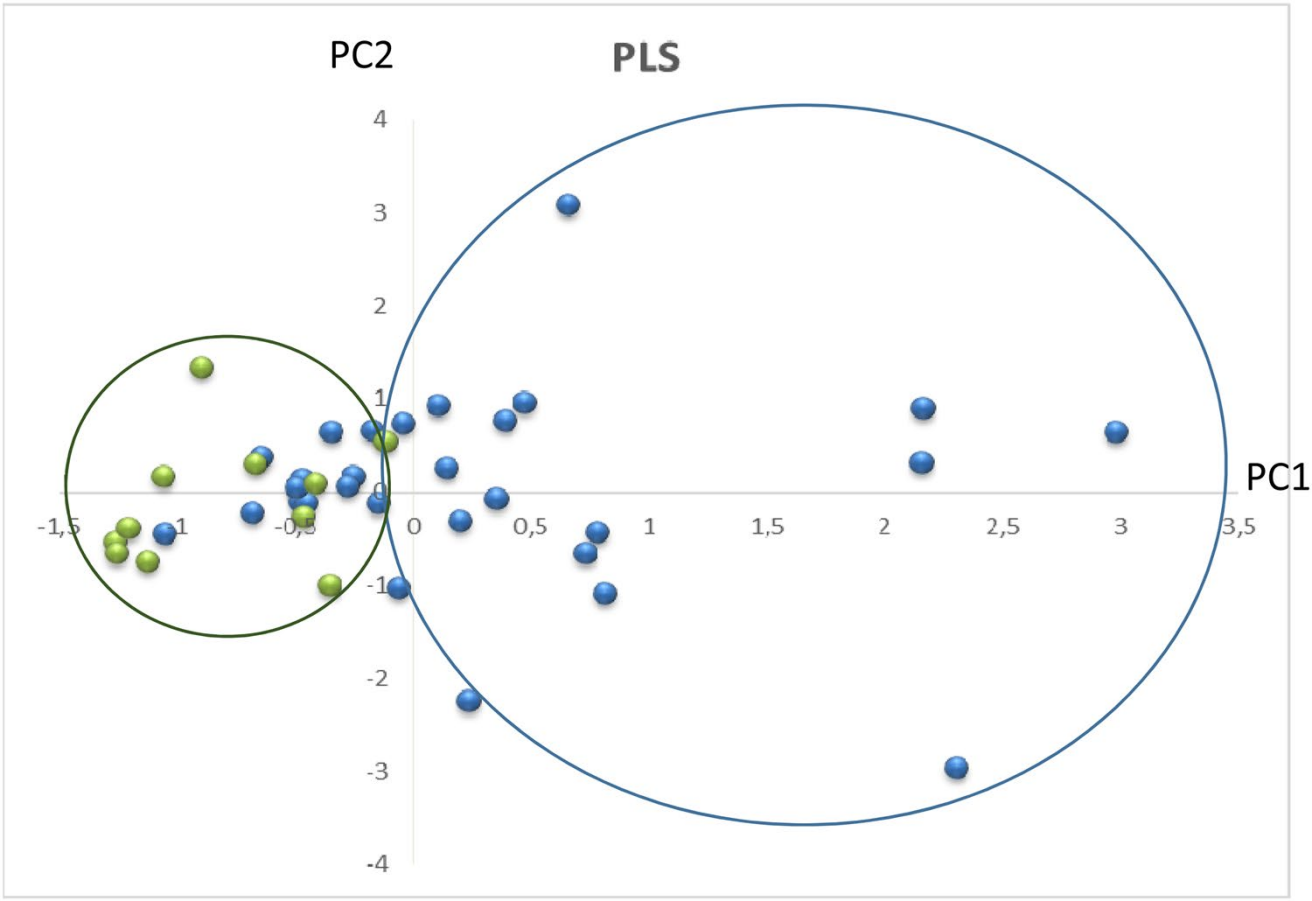
PLS score plot represents differential distribution between AD and healthy control groups. As visual approach, blue circle englobes AD patients, green circle englobes healthy and some AD participants. PC: principal component.

Different combinations of the previously selected compounds (myo-inositol, creatine, glutamine, acetylcholine) were used to carry out multivariate analysis. In general, the developed models showed AUC-ROC values between 0.382 and 0.806 (see Table 4). However, model 1 (myo-inositol, creatine, glutamine, acetylcholine) showed best performance. The results from this multivariate analysis model are depicted in Fig. 2. As can be seen the salivary levels of myo-inositol, creatine, glutamine and acetylcholine could discriminate between AD and HC groups, except for a 30% of doubtful cases. Most of these not clearly classified cases showed the lowest CDR scores (0–0.5). The model showed an AUC-ROC of 0.806 (CI 95%, 0.674–0.939). Regarding the predictive power of this model it is important to highlight the specificity of 92%, while the sensitivity was 61%.

**Table 4:** Diagnosis indices for the developed multivariate analysis.

Model	Index (CI 95%)						
	AUC-ROC	Sensitivity	Specificity	Positive predictive value	Negative predictive value	Positive odds ratio	Negative odds ratio
1	0.806 (0.674, 0.939)	61.3 (42.3, 77.6)	91.7 (59.7, 99.6)	95.0 (73.1, 99.7)	47.8 (27.4, 68.9)	7.35 (1.10, 49.04)	0.42 (0.26, 0.68)
2	0.632 (0.445, 0.818)	69.7 (52.7, 82.6)	75.0 (46.8, 91.1)	88.5 (71.0, 96.0)	47.4 (27.3, 68.3)	2.79 (1.02, 7.62)	0.40 (0.23, 0.71)
3	0.602 (0.405, 0.800)	73.3 (55.6, 85.8)	66.7 (39.1, 86.2)	84.6 (66.5, 93.9)	50.0 (28.0, 72.0)	2.20 (0.96, 5.04)	0.40 (0.21, 0.77)
4	0.386 (0.221, 0.551)	32.3 (18.6, 49.9)	83.3 (55.2, 95.3)	83.3 (55.2, 95.3)	32.3 (18.9, 49.9)	1.94 (0.49, 7.57)	0.81 (0.57, 1.15)
5	0.767 (0.606, 0.929)	83.9 (67.4, 92.9)	66.7 (39.1, 86.2)	86.7 (70.3, 94.7)	61.5 (35.5, 82.3)	2.52 (1.11, 5.68)	0.24 (0.10, 0.56)
6	0.762 (0.604, 0.920)	71.0 (53.4, 83.9)	75.0 (46.8, 91.1)	88.0 (70.0, 95.8)	50.0 (29.0, 71.0)	2.84 (1.04, 7.76)	0.39 (0.21, 0.70)
7	0.571 (0.386, 0.756)	25.8 (13.7, 43.2)	91.7 (64.6, 98.5)	88.9 (56.5, 98.0)	32.4 (19.1, 49.2)	3.10 (0.43, 22.19)	0.81 (0.61, 1.08)
8	0.676 (0.489, 0.863)	80.6 (63.7, 90.8)	58.3 (32.0, 80.7)	83.3 (66.4, 92.7)	53.8 (29.1, 76.8)	1.94 (0.97, 3.86)	0.33 (0.15, 0.72)
9	0.548 (0.357, 0.740)	29.0 (16.1, 46.6)	91.7 (64.6, 98.5)	90.0 (59.6, 98.2)	33.3 (19.8, 50.4)	3.48 (0.49, 24.62)	0.77 (0.58, 1.04)
10	0.692 (0.515, 0.869)	58.1 (40.8, 73.6)	75.0 (46.8, 91.1)	85.7 (65.4, 95.0)	40.9 (23.3, 61.3)	2.32 (0.83, 6.47)	0.56 (0.35, 0.91)
11	0.738 (0.583, 0.892)	58.1 (40.8, 73.6)	91.7 (64.6, 98.5)	94.7 (75.4, 99.1)	45.8 (27.9, 64.9)	6.97 (1.04, 46.60)	0.46 (0.30, 0.71)

(Model 1) Myo-inositol, glutamine, creatine, acetylcholine; (Model 2) Myo-inositol, creatine; (Model 3) Myo-inositol, glutamine; (Model 4) Myo-inositol, acetylcholine; (Model 5) Glutamine, creatine; (Model 6) Glutamine, acetylcholine; (Model 7) Creatine, acetylcholine; (Model 8) Myo-inositol, creatine, glutamine; (Model 9) Myo-inositol, creatine, acetylcholine; (Model 10) Myo-inositol, glutamine, acetylcholine; (Model 11) Glutamine, creatine, acetylcholine.

CI: confidence interval, AUC-ROC: Area Under Curve- Receiver Operating Curve.

## Discussion

Saliva sample has the advantage of simple collection, being convenient and acceptable for all patients, and achieving high participant recruitment. In addition, several studies have found that saliva levels were comparable to blood levels, adding some clinical value to these salivary determinations^39^. The salivary levels of neurotransmitters were evaluated from two different approaches. First, univariate analysis showed significant differences between AD and HC participants for some compounds (myo-inositol, creatine, acetylcholine), as well as, correlations with clinical AD variables (neuropsychological scales, CSF biomarkers) for other compounds (myo-inositol, glutamine, creatine). Second, multivariate analysis, including the previously selected compounds (myo-inositol, glutamine, creatine, acetylcholine), showed satisfactory accuracy discriminating between AD and healthy control individuals.

Regarding univariate analysis, glutamine showed negative correlation with MMSE score. Similarly, previous studies showed increased CSF levels of glutamate and glutamine in patients with probable AD^24^. In addition, the glutamate/glutamine ratio showed a reduction in ageing and AD^40^. It could be explained by the glutamine neuroprotector effect observed in cell culture, protecting against amyloid-β peptide^41^. Also, in the present study myo-inositol levels were lower in AD patients. Specifically, salivary myo-inositol showed lower levels in saliva samples from patients suffering from important cognitive impairment, while higher salivary levels corresponded to higher neuropsychological tests scores (RBANS, MMSE). In this sense, Shinno et al. found lower levels of myo-inositol in brain, specifically in the anterior cingulate gyros, and it could be associated with AD development^42^. In contrast, a previous study found that urinary myo-inositol excretion could be used as cognitive impairment biomarker^43^. In definitive, myo-inositol levels could be impaired under AD conditions, making difficult the transport of substances between brain and other biofluids^44^. In addition, salivary myo-inositol showed correlation with CSF β-amyloid levels, considered the gold standard in AD diagnosis. Similarly, previous studies showed that brain myo-inositol measured by magnetic resonance spectroscopy correlated with β-amyloid even before the cognitive impairment appearance^45^. In general, these findings could constitute a relevant aspect in non-invasive diagnosis development, since these amino acids and derivates measured in non-invasive samples (saliva, urine) could replace CSF sampling in AD current diagnosis. Moreover, other inositol stereoisomers, such as scyllo-inositol, had been tested as potential therapy because of its anti-oligomer activity in cell culture^46^. In this sense, an increase in myo-inositol levels could be protective against amyloid-β plaques formation and so AD development.

For acetylcholine, the present study showed higher levels in patients with lower scores for RBANS and higher scores for CDR and FAQ. Of note, CDR and FAQ lower scores show better cognitive status, while higher punctuations in RBANS show better cognitive state. Therefore, high salivary acetylcholine levels corresponded to patients with cognitive impairment. Nevertheless, opposite results were obtained in a previous study carried out in plasma samples^47^.

For creatine, lower salivary levels were obtained in AD patients, as well as positive correlation with MMSE. In this sense, a few studies in literature have focused on this metabolite as neurodegeneration biomarker^48,49^.

Regarding multivariate analysis, the studied neurotransmitters could be useful as AD biomarkers. Specifically, the simultaneous determination of a panel of 4 neurotransmitters (myo-inositol, acetylcholine, creatine, glutamine) could improve the accuracy differentiating between AD and healthy participants. In general, these results would explain an alteration in neurotransmission under AD conditions. A previous study in AD showed an imbalance in neurotransmitters levels in brain^50^. In addition, neurotransmitters’ transporters showed alterations in the pathology^51^. Therefore, neurotransmitters were accumulated or reduced in brain and different biofluids depending on their transport, and an alteration in their distribution could lead to the typical manifestations of AD. In this sense, a previous AD study determining glutamate in CSF samples showed sensitivity 95.2%, specificity 100%, and AUC = 0.99^24^. Also, a study determining 10 amino acids as potential blood biomarkers in AD showed an AUC = 0.948^52^. Despite obtaining satisfactory diagnosis indexes, these works used invasive samples. Nevertheless, the present study could allow a first screening approach in early AD diagnosis, using non-invasive samples. In fact, this new tool, showing high positive predictive value, could reduce invasive and expensive diagnostic methods (CSF biomarkers, neuroimaging techniques) only to doubtful cases, avoiding conventional diagnosis methods in positive screening cases.

Regarding specificity, some studies found neurotransmitter impairment in other dementias (Parkinson disease, vascular dementia, dementia with Lewy bodies, fronto-temporal dementia)^53,54^, or pathologies^55^. Nevertheless, there is a lack of studies focusing on differential AD diagnosis based on neurotransmitters.

Among the study limitations, it is important to highlight the small sample size and so the reduced statistical power. Also, a single internal standard has been used to correct 10 different analytes which could impact in quantitative accuracy. In addition, some saliva collection variables have not been standardised, and potential biomarkers specificity has not been evaluated.

## Conclusions

Some neurotransmitters levels in saliva could be impaired under AD conditions. Among the evaluated compounds, it is important to highlight myo-inositol, acetylcholine, creatine and glutamine, since these compounds correlated with cognitive impairment associated to AD, and also myo-inositol showed correlation with CSF amyloid-β levels. These neurotransmitters could be used as promising non-invasive biomarkers for AD. From them, an optimum multivariate model was developed constituting a potential tool in the early screening of AD patients and reducing invasive and expensive diagnostic methods, which would be applied to doubtful cases. Nevertheless, further work is required, increasing sample size to clinically validate these preliminary results, as well as to evaluate their specificity.

## Supplementary information

Supplementary file1

Supplementary file2

Supplementary file3
