# Supplementary material for: Early neurotransmission impairment in non-invasive Alzheimer Disease detection
Source: Sci Rep. 2020 Oct 2;10:16396. doi: 10.1038/s41598-020-73362-z (PMC7532202; doi:10.1038/s41598-020-73362-z)
Supplement: Supplementary file 1 — Supplementary file1 [file 41598_2020_73362_MOESM1_ESM.docx]

**Early neurotransmission impairment in non-invasive Alzheimer Disease detection**

Carmen PEÑA-BAUTISTA ^1^, Isabel TORRES-CUEVAS ^1^, Miguel BAQUERO ^2^, Inés FERRER ^2^, Lorena GARCÍA ^2^, Máximo VENTO ^1^, Consuelo CHÁFER-PERICÁS ^1,*^

^1^Neonatal Research Unit, Health Research Institute La Fe, Valencia, Spain

^2^Neurology Unit, University and Polytechnic Hospital La Fe, Valencia, Spain

**Figure S1.** Plots representing correlation results between metabolites and neuropsychological measures
