# Supplementary material for: Early neurotransmission impairment in non-invasive Alzheimer Disease detection
Source: Sci Rep. 2020 Oct 2;10:16396. doi: 10.1038/s41598-020-73362-z (PMC7532202; doi:10.1038/s41598-020-73362-z)
Supplement: Supplementary file 2 — Supplementary file2 [file 41598_2020_73362_MOESM2_ESM.docx]

**Early neurotransmission impairment in non-invasive Alzheimer Disease detection**

Carmen PEÑA-BAUTISTA ^1^, Isabel TORRES-CUEVAS ^1^, Miguel BAQUERO ^2^, Inés FERRER ^2^, Lorena GARCÍA ^2^, Máximo VENTO ^1^, Consuelo CHÁFER-PERICÁS ^1,*^

^1^Neonatal Research Unit, Health Research Institute La Fe, Valencia, Spain

^2^Neurology Unit, University and Polytechnic Hospital La Fe, Valencia, Spain

**Table S1.** Analytical characteristics of the developed method

| Analyte | Retention Time  (min)^a^ | Linearity  Calibration equation  y = a+þ*x  R^2^ | LOD  (nmol L^-1^) | LOQ  (nmol L^-1^) | Precision RSD (%)^b^ | |
| --- | --- | --- | --- | --- | --- | --- |
|  |  |  |  |  | Intra-day  (n= 3) | Inter-day (n= 6) |
| Taurine | 0.75 ± 0.05 | a± s_a_ = 0.031 ± 0.018  b ± s_b_ =0.0001 ± 0.0000  R^2^= 0.999 | 0.09 | 0.3 | 2.9 | 6.2 |
| NAA | 0.62 ± 0.03 | a± s_a_ =-0.007 ± 0.016  b ± s_b_ =0.0004 ± 0.0001  R^2^= 0.998 | 0.3 | 1.2 | 2.1 | 5.9 |
| Myo-inositol | 0.74±0.07 | a± s_a_ =0.0004 ± 0.0001  b ± s_b_ =0.0000007 ± 0.0000001  R^2^= 0.996 | 0.3 | 1.2 | 1.9 | 4.3 |
| Aspartic Acid | 0.66± 0.04 | a± s_a_ =0.0001 ± 0.0012  b ± s_b_ =0.0000233 ± 0.0000001  R^2^= 0.997 | 0.3 | 1.2 | 3.2 | 7.0 |
| Glutamic Acid | 1.01 ± 0.05 | a± s_a_ =0.13 ±0.08  b ± s_b_ =0.0036 ± 0.0001  R^2^= 0.999 | 0.3 | 1.2 | 0.8 | 4.3 |
| Glutamine | 1.13 ± 0.06 | a± s_a_ =0.013 ± 0.002  b ± s_b_ =0.00030 ± 0.00001  R^2^= 0.999 | 0.3 | 1.2 | 1.4 | 8.2 |
| GABA | 1.50 ± 0.05 | a± s_a_ =0.020 ± 0.009  b ± s_b_ =0.00043 ± 0.00001  R^2^= 0.997 | 1.4 | 4.6 | 3.0 | 10.2 |
| Acetylcholine | 1.72±0.01 | a± s_a_ =0.1 ± 0.8  b ± s_b_ =0.0562 ± 0.0005  R^2^= 0.999 | 0.09 | 0.3 | 2.5 | 8.4 |
| Creatine | 1.19 ± 0.03 | a± s_a_ =0.24 ± 0.13  b ± s_b_ =0.0042 ± 0.0001  R^2^= 0.999 | 0.09 | 0.3 | 1.3 | 2.9 |

NAA: N-Acetyl-L-aspartic acid; s: standard deviation; LOD: Limit of detection; LOQ: Limit of quantification

^a^: mean values ± standard deviation

^b^: Calculated at a concentration of 1000 nmol L^-1^ for each analyte.
