# Supplementary material for: Early neurotransmission impairment in non-invasive Alzheimer Disease detection
Source: Sci Rep. 2020 Oct 2;10:16396. doi: 10.1038/s41598-020-73362-z (PMC7532202; doi:10.1038/s41598-020-73362-z)
Supplement: Supplementary file 3 — Supplementary file3 [file 41598_2020_73362_MOESM3_ESM.docx]

**Early neurotransmission impairment in non-invasive Alzheimer Disease detection**

Carmen PEÑA-BAUTISTA ^1^, Isabel TORRES-CUEVAS ^1^, Miguel BAQUERO ^2^, Inés FERRER ^2^, Lorena GARCÍA ^2^, Máximo VENTO ^1^, Consuelo CHÁFER-PERICÁS ^1,*^

^1^Neonatal Research Unit, Health Research Institute La Fe, Valencia, Spain

^2^Neurology Unit, University and Polytechnic Hospital La Fe, Valencia, Spain

**Table S2.** Extraction recoveries of analytes from the analysis of spiked saliva samples at different concentrations

| Analyte | Low^a^  (500 nmol L^-1^, n= 3) | Medium^a^  (1000 nmol L^-1^, n= 3) | High^a^  (2000 nmol L^-1^, n= 3) |
| --- | --- | --- | --- |
| Taurine | 94±13 | 115±6 | 108±4 |
| NAA | 109±5 | 113±5 | 105±3 |
| Myo-inositol | 112± 11 | 111±4 | 107±8 |
| Aspartic acid | 101±3 | 101± 4 | 100±2 |
| Glutamic acid | 108±1 | 117±15 | 103±6 |
| Glutamine | 94±2 | 118±9 | 106±6 |
| GABA | 98±4 | 116±3 | 101±1 |
| Acetylcholine | 87±5 | 115±3 | 121±8 |
| Creatine | 83±10 | 115±6 | 120±3 |

^a^: mean values ± standard deviation
